# The LIFE STREAMS Project for the Recovery of the Native Mediterranean Trout in Six Italian Pilot Areas: Planning and Adoption of Conservation Actions

**DOI:** 10.3390/biology14050573

**Published:** 2025-05-20

**Authors:** Antonella Carosi, Lorenzo Talarico, Claudia Greco, Antonia Vecchiotti, Susanna D’Antoni, Alessandro Longobardi, Stefano Macchio, Marco Carafa, Paolo Casula, Antonio Perfetti, Paola Amprimo, Alessandro Rossetti, Federico Morandi, Davide Alberti, Pietro Serroni, Stefano Raimondi, Diego Mattioli, Nadia Mucci, Massimo Lorenzoni

**Affiliations:** 1 Department of Chemistry, Biology and Biotechnologies, University of Perugia, 06123 Perugia, Italy; massimo.lorenzoni@unipg.it; 2 Department of Biology, Tor Vergata University of Rome, 00133 Rome, Italy; 3 Institute for Environmental Protection and Research (ISPRA), 00144 Rome, Italy; claudia.greco@isprambiente.it (C.G.); susanna.dantoni@isprambiente.it (S.D.); alessandro.longobardi@isprambiente.it (A.L.); stefano.macchio@isprambiente.it (S.M.); nadia.mucci@isprambiente.it (N.M.); 4 Ufficio Gestione e Monitoraggio Biodiversità, Parco Nazionale della Maiella, 67039 Sulmona, Italymarco.carafa@parcomajella.it (M.C.); 5 Servizio Tecnico Forestale, Agenzia Forestale Regionale per lo Sviluppo del Territorio e dell’Ambiente della Sardegna (Forestas), 09033 Cagliari, Italy; pcasula@forestas.it; 6 Regione Liguria, Direzione Generale Turismo, Agricoltura e Aree Protette, 16133 Genova, Italy; antonio.perfetti@regione.liguria.it; 7 Ente Parco Regionale Montemarcello-Magra-Vara, 19038 Sarzana, Italy; p.amprimo@parcomagra.it; 8 Servizio Gestione del Territorio e Sviluppo Sostenibile, Parco Nazionale dei Monti Sibillini, 62039 Visso, Italy; rossetti@sibillini.net (A.R.); federico.morandi@sibillini.net (F.M.); 9 Servizio Promozione, Conservazione, Ricerca e Divulgazione della Natura, Parco Nazionale delle Foreste Casentinesi Monte Falterona e Campigna, 52015 Pratovecchio, Italy; davide.alberti@parcoforestecasentinesi.it; 10 Area Conservazione, Studi e Ricerche, Parco Nazionale del Pollino, 85048 Rotonda, Italy; pietro.serroni@parconazionalepollino.it; 11 Aree Protette e Biodiversità, Legambiente Onlus, 00118 Rome, Italy; s.raimondi@legambiente.it; 12 Noesis European Development Consulting, 06034 Foligno, Italy; d.mattioli@noesisonline.eu

**Keywords:** Mediterranean trout, Italian peninsula, endangered species conservation, biological invasions, genetic introgression, protected areas, fish management

## Abstract

The Mediterranean trout (currently referred to as *Salmo ghigii* for Corsican and Italian-native populations) is listed as Endangered in the IUCN Red List, due to fragmented distribution and declining populations across its whole range, and is included in Annex II of the European Habitat Directive. The widespread genome introgression from the invasive Atlantic trout (*Salmo trutta*), overexploitation, and habitat alterations represent major threats to the persistence of native populations. The LIFE18NAT/IT/000931 STREAMS project aims to enhance conservation status of Mediterranean trout in 6 Italian pilot areas (Maiella, Sibillini Mountains, Casentino Forests and Pollino National Parks, Montemarcello-Magra-Vara Regional Park, and Sardinia with five sites of the Natura 2000 Network), and in 19 transferability areas covering almost the whole Italian species range. To achieve this, the following conservation strategies were implemented: (i) the identification of residual native populations; (ii) eradication of entirely Atlantic-exotic populations and removal of hybrids in admixed populations; (iii) restocking/reintroduction of native populations; (iv) monitoring/improving the Mediterranean trout habitats quality; (v) production of the “Guidelines for the conservation and management of native Mediterranean trout and its habitat”; and (vi) the prevention of illegal stocking. Here, we present the project rationale, major outcomes on demographic and genetic characterization of wild populations, and summary results from conservation actions.

## Simple Summary

The Mediterranean trout *Salmo ghigii* is listed as Endangered on the IUCN Red List and included in Annex II of the European Habitat Directive. The extensive genetic pollution due to the introduction of th invasive Atlantic trout (*Salmo trutta*) and habitat degradation are among the main threats to the survival of the species. Our aims were as follows: (i) to offer a comprehensive overview of the current conservation status of the species across six Italian pilot areas (National or Regional Parks and Nature 2000 sites), based on the research conducted within the LIFE18NAT/IT/000931 STREAMS project; (ii) show the summary results achieved through a series of combined actions, aimed at enhancing the conservation status of the species within a broad and heterogeneous spatial context. A national conservation strategy for the Mediterranean trout was addressed, intending to provide a management framework encompassing the species range. Our results underscore the relevance of a multilevel genetic–ecological approach for the preservation of intensively managed taxa, such as salmonids, which could be extended to other areas and species subject to similar management issues. Moreover, our research emphasizes the key role of protected areas in the detection, recovery, and maintenance of residual native populations, which are not exempt from the effects of human-induced stressors.

## 1. Introduction

**Figure 1:**
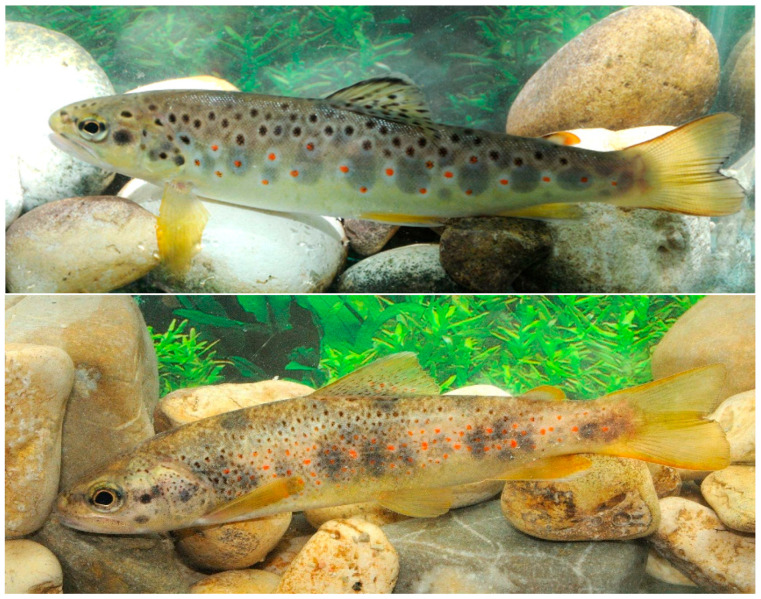
Pictures of brown trout, *Salmo trutta* (**above**), and Mediterranean trout, *Salmo ghigii* (**below**) (Photo credits: Massimo Lorenzoni).

The Mediterranean trout is a taxon of the *Salmo trutta* species complex [1]. In a context of debated and unresolved *Salmo* taxonomy and systematic [2,3,4,5,6] (Figure 1), the current nomenclature assigns *Salmo ghigii* Pomini, 1941, to the Italian peninsular and native Corsican and Sardinian populations, replacing the former attribution to *Salmo cettii* Rafinesque, 1810 [5,6]. This species is essential for the functioning of aquatic ecosystems, especially in mountain streams where it is usually the dominant species [7], an apical predator [4] and a pivotal ecological indicator [8,9]. In addition, the Mediterranean trout is also a species of great interest for recreational fishing. Despite the high ecological and cultural value, numerous interacting stressors threaten the persistence of the species, whose populations are often fragmented, patchily distributed or isolated. Undergoing population declines and narrowing distribution across the whole species range has justified its classification as Endangered with a declining population trend, according to the IUCN [10], and Critically Endangered by the IUCN Red List of Italian vertebrates [11]. The Mediterranean trout is also included in Annex II of the European Habitat Directive 92/43/EEC, which identifies species requiring the establishment of special areas for their conservation. The fourth monitoring report of the Habitat Directive indicates that Italian populations are experiencing an unfavourable–bad conservation status with a declining trend [12]. The extensive human-mediated genome introgression from alien lineages, along with alterations in water flow, pollution, overexploitation, habitat alterations, and climate change, are believed to be major threats to the survival of wild native populations [13,14,15,16,17].

To supply wild populations subject to overfishing (e.g., recreational angling), large-scale stocking, both authorized and illegal, has been reiterated since 1850 [6]. This involved the widespread introduction of hatchery-reared alien Atlantic trout (*Salmo trutta* Linnaeus, 1758 *sensu stricto*; Figure 1, leading to massive hybridization between native and domestic trout lineages in the wild [13,14,17,18]. the Atlantic trout is indeed one of the 100 World’s Worst Invasive Alien Species because of its remarkable impact on native biological diversity [19]. As a consequence, the Mediterranean trout is currently considered extinct in most of its Italian range and is supplanted by populations of non-native trout or their hybrids [13]. The conservation status of native populations has been further deteriorated by the relatively recent stocking with commercial “Mediterranean trout” of uncertain genetic integrity and origin [20].

In recent decades, a number of European-funded projects have aimed to improve the conservation status of native salmonids and their habitats, particularly within Italian-protected areas and Natura 2000 sites (i.e., INTERREG ShareSalmo, LIFE Nat.Sal.Mo, HydroLife, LIFE+ BIOAQUAE, LIFE+ TROTA, LIFE GRAYMARBLE) that should play a key role in freshwater (fish) biodiversity conservation and habitat restoration [21,22,23,24]. Many of them envisaged the implementation and successful application of various conservation measures, such as the eradication of exotic populations, removal of hybrids in admixed populations, reintroduction of native trout, and fishing regulations [25,26,27,28,29]. Similarly, stocking with fry from native stocks and/or by translocations of wild native individuals have been reported to be successful in brown trout populations in the northern French Alps, resulting in a significant decrease in the percentages of non-native alleles [30,31]. On the other hand, the unsuccessful artificial reproduction of Corsican native trout was reported in the LIFE TRUITE project.

**Figure 2:**
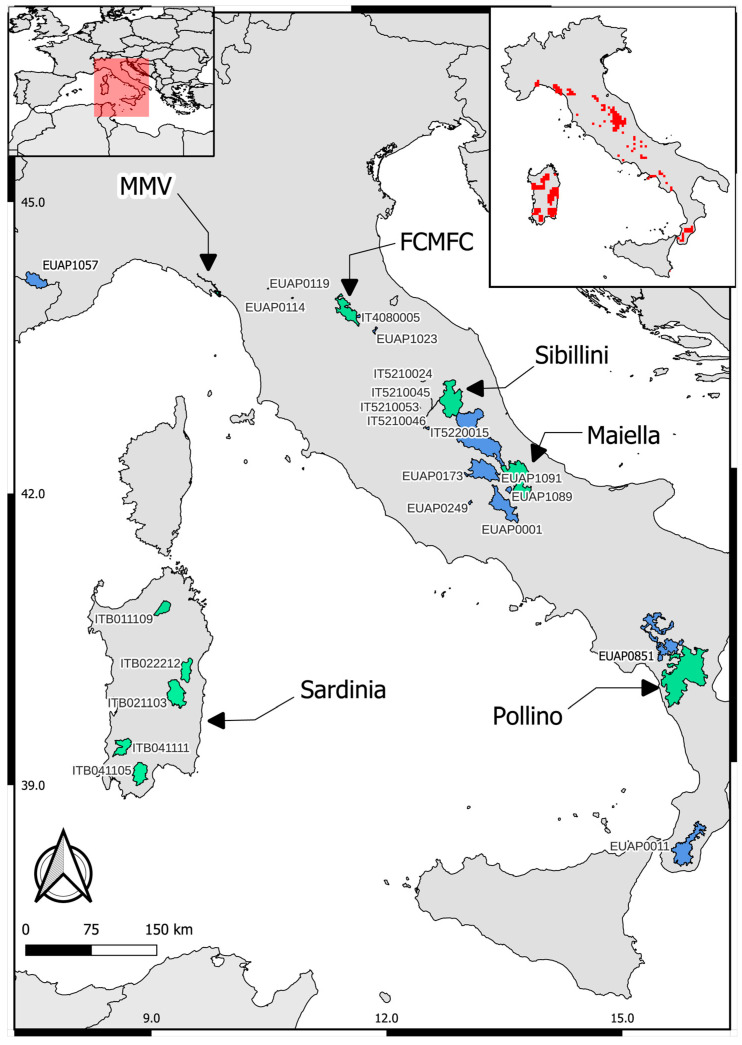
Spatial distribution of 6 pilot protected areas in Italy (in aquamarine, indicated by arrows) and 19 transferability areas (in pale blue). The top-right map shows the current Italian distribution of the Mediterranean trout (red area).

However, none of the previously mentioned projects covered the whole distribution range of the target species, and many authors outlined the lack of a comprehensive conservation strategy for the Mediterranean trout, the insufficient coordination of management efforts, and the fragmentation of the sparse information available on the current species and habitat status assessment [9,32]. Within this context, the LIFE18NAT/IT/000931 STREAMS (*Salmo ceTtii* REcovery Actions in Mediterranean Streams) project (2019–2025) aims at enhancing the conservation status of Mediterranean trout across (almost) its whole Italian distribution, focusing on 6 protected pilot areas (Maiella, Sibillini Mountains, Casentino Forests, and Pollino National Parks, Montemarcello-Magra-Vara Regional Park, and 5 Natura 2000 sites in Sardinia) plus 19 designated transferability areas (3 National parks, 6 Natural reserves, 3 Regional parks, and 7 Special conservation areas) in which the knowledge gained from the project’s outcomes and insights can be replicated (Figure 2). The LIFE STREAMS Project was designed in the wake of the LIFE12 NAT/IT/000940 TROTA–namely the Trout Population Recovery in Central Italy project that focused on the recovery and conservation of Mediterranean trout in Central Italy–seeking to develop and implement a comprehensive management strategy across the entire native range of the species to enhance its conservation status through a series of combined actions characterized by a multidisciplinary approach. Among known anthropogenic threats to the persistence of Mediterranean trout, the project primarily focused on reducing genetic introgression from alien strains, improving the quality of freshwater habitats by mitigating river fragmentation, and reducing illegal stocking. Specifically, the conservation strategy includes the following actions: (i) the identification of residual native populations; (ii) the removal of Atlantic-exotic populations and hybrid/Atlantic specimens; (iii) restocking and reintroduction of native populations, through the production in hatcheries of genetically selected fertilized eggs and/or alevins; (iv) monitoring and improving the quality of the Mediterranean trout habitats, through the application of the Ecological Flow and the reduction in the river fragmentation, following the European Water Framework Directive 2000/60/EC, the European Biodiversity Strategy and the Nature Restoration Law; (v) production of the “Guidelines for the conservation and management of native Mediterranean trout and its habitat”; and (vi) developing an effective strategy to prevent illegal stocking. After the completion of the preparatory actions of the project, including the genetic and ecological characterization of wild Mediterranean trout populations and habitat, the project is ongoing with the implementation of concrete conservation actions on selected suitable sites.

In this study, we achieve the following: (i) describe the workflow of the project that relies on an integrated genetic-demographic decisional approach to determine site-specific conservation action(s); (ii) provide a picture of the current conservation status of wild trout populations in the investigated areas as resulting from the project’s preliminary actions; (iii) provide the results obtained to date in concrete conservation actions; and (iv) highlight the strengths and weaknesses of the project.

## 2. Materials and Methods

### 2.1. Study Area

We selected six pilot areas (Figure 2)–Maiella National Park (hereafter “Maiella”), Pollino National Park (“Pollino”), 2 SACs and 3 SPAs in the Sardinia region (collectively referred to as “Sardinia”), the Casentino Forests National Park (“FCMFC”), Montemarcello-Magra-Vara Natural Regional Park (“MMV”), and Sibillini Mountains National Park (“Sibillini”)–considering the following features: they were representative of heterogeneous environments (e.g., climate, lithology, hydrology) across the whole native range of Mediterranean trout in Italy; were exposed to various anthropic impact and socio-economic features; fell within protected zones including numerous Natura 2000 Sites noted for conservation actions; and have available background knowledge on the conservation (genetic) status of wild trout populations. Information on the environmental diversity (elevation, extension, hydrography, lithology), background information on the genetic status of trout populations, and local-specific threats to the conservation of Mediterranean trout are all summarized in Table 1 for each area. In brief, both hilly and mountain sites were investigated, hosting native, admixed, or entirely alien brown trout populations, based on the information that was available at the start of the project.

### 2.2. Field Sampling and Data Collection

Field activities within the preparatory actions were carried out in 84 sites (a single sample each) from 70 watercourses between June and November 2020. We collected environmental parameters primarily affecting fish distribution: altitude, pH, flow rate, electric conductivity, dissolved oxygen and water temperature. For details on data collection, see Carosi et al. [15] and Supplementary Material File S1. Fish were captured by electrofishing, following the two-pass removal method [40,41] and annotating abundance and biomass for each taxon. Brown trout were temporarily anaesthetized with a water solution containing 10–35 mg/L of tricaine methanesulfonate (MS-222) [18,42], to facilitate the collection of individual biometrics (weight and total length) and biopsies (10–15 scales for age determination in the laboratory, and a fin clip for DNA extraction) before releasing into the wild. Sampling activities followed standard procedures and were approved by relevant authorities for each pilot area.

### 2.3. Environmental and Demographic Assessment of Wild Populations

For each population, we estimated the following:

- Density (ind m^−2^) and standing crop (g m^−2^);
- Age structure (age individually attributed by the scalimetric method);
- Average individual total length;
- Average individual relative weight (Wr), a measure of body condition whose values in the range 95–105 indicate optimal conditions [43];
- Proportional stock density index (PSD), ranging between 0 and 100, that estimates population structure deviations from a hypothetical balanced population, i.e., 35 ≤ PSD ≤ 65 [44]. Further details on PSD and Wr can be seen in the work of Carosi et al. [15] and in Supplementary Material File S1.

We used boxplots to examine patterns of demographic measures and abiotic/environmental variables across target areas, evaluating overall and area-pairwise statistical differences through Kruskal–Wallis and Mann–Whitney U tests (adjusting *p*-values with Bonferroni correction for multiple testing), respectively, as implemented in PAST 4.1 [45].

We also performed a Principal Components Analysis in PAST 4.1 to visually inspect overall sampling site heterogeneity, while assessing relationships among considered demographic/abiotic/environmental variables–to account for different scales among variables, each variable was normalized (dividing by its standard deviation).

### 2.4. Preliminary Genetic Assessment of Wild Populations

We typed individuals at the nuclear LDH-C1 gene and a 535 bp sequence of the mitochondrial D-loop for the preliminary genetic characterization of populations. Both markers are routinely used for a rapid, economically convenient and reliable assessment of hatchery introgression in wild trout populations in Italy as they unequivocally allow distinguishing between Mediterranean native and Atlantic alien alleles/haplotypes [17,18,32,46,47]. Laboratory procedures (DNA extraction, PCR amplification, genotyping of LDH-C1 and sequencing of the D-loop fragment) followed protocols described in Padula et al. [46] and Talarico et al. [47]. By combining LDH-C1 and D-loop profiles, we classified individuals into three genetic classes: “native” for individuals showing native profiles at the two markers; “alien” for individuals revealing Atlantic (hatchery) profiles at both LDH-C1 and D-loop; “introgressed” for individuals with admixed native-alien profiles (for further details, see Talarico et al. [47]). The population composition, in terms of frequency of genetic classes, was used to assist decision making for downstream conservation actions (see Figure 2 and “decisional workflow” section).

### 2.5. Advanced Genetic Characterization to Define Population Structure and Identify Putative Spawners

Based on outcomes from the preliminary genetic characterization of wild populations, we aimed at identifying relatively pure-native wild population(s) within each pilot area to serve as source(s) of spawners and/or transferable individuals for population enhancement purposes (see Figure 2 and “decisional workflow” section). Therefore, we captured a various, demographically sustainable number of individuals from selected populations that were individually tagged (see “Production of native eggs and alevins in captive condition” paragraph for details) and temporarily hosted within hatcheries until the completion of advanced genetic characterization. When native wild populations were not found, we genetically characterized additional wild populations from different sites/watercourses in the neighborhood or hatchery broodstock of local Mediterranean trout strains. In brief, since Mediterranean trout populations often show strong population differentiation even at the local spatial scale [17,32,48], and population enhancement by genetically dissimilar eggs/fries should be avoided especially for conservation programs [17], we performed an advanced genetic characterization aimed at the following: defining genetic population structure and, in turn, identifying Management Units (MU) [49] within each pilot area; selecting putative spawners by accurately assessing their introgression degree. To carry this out, putative spawners–along with 107 reference domestic Atlantic samples from Italian hatcheries and local reference of wild native trout (when available)–were typed at LDH-C1, D-loop plus 15 microsatellite loci (see Talarico et al. [47] for details on amplification and genotyping protocols). Retained suitable spawners had native LDH-C1/D-loop profiles coupled with <2% introgression from Atlantic strains–as assessed through a Bayesian population structure analysis performed with the STRUCTURE v. 2.3.4 software [50] for each pilot area separately–and their offspring was used to restock target wild populations within the same MU.

### 2.6. Decisional Workflow for Conservation Actions

**Figure 3:**
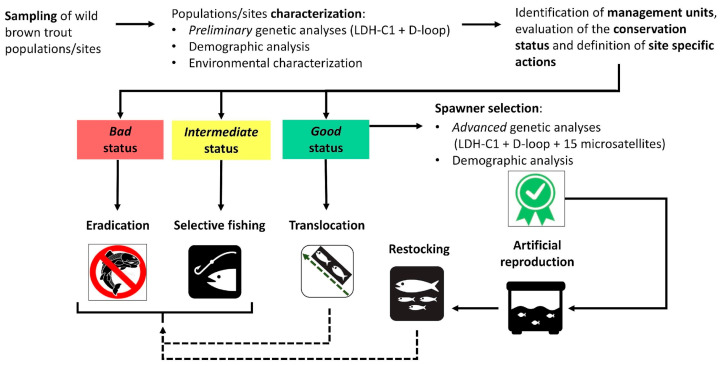
Decisional workflow for conservation actions on wild (Mediterranean) brown trout populations based on the adopted genetic-demographic approach.

The decisional workflow is summarized in Figure 3. In the first step, we assessed the conservation status of wild populations based on an integrated genetic (i.e., the preliminary characterization described above) and demographic approach, categorizing them as follows: (i) bad status, when the population was composed mainly of alien and/or hybrid individuals, and these sites were considered potentially suitable for eradication programs; (ii) intermediate status, when the population was partially introgressed, for which we recommended selective fishing (i.e., experimental angling aimed at selectively removing hybrids and alien caught trout based on morphological screening); (iii) good status, when the population preserved a high degree of genetic integrity and were eligible as a source of individuals for translocations activities or for the spawner selection/artificial reproduction in hatcheries. Ecological and demographic analysis of the trout populations, combined with the results of the environmental characterization, played a key role in the site’s selection for the implementation of concrete conservation actions. To enhance project outcomes while preserving the maintenance of native wild trout populations, only sites meeting specific demographic and environmental requirements were selected among deemed genetically suitable sites. Several criteria guided the multi-level selection process: (i) the locations designated for spawner collection needed to exhibit high spawner abundance, significant population density, and an optimal age structure; (ii) the sites identified for supportive breeding and selective fishing were required to have appropriate environmental conditions for trout fishing, optimal density, a balanced age structure with young of the year adequately represented; and (iii) the areas selected for the alien individuals’ eradication and the reintroduction of Mediterranean trout had to be of modest size in terms of length and width, and isolated from adjacent areas to prevent recolonization by alien trout, entirely accessible for electrofishing purposes and facilitates the removal and transportation of trout, while also providing suitable environmental conditions for trout, particularly concerning summer water flows [51].

### 2.7. Planned Conservation Strategies

We included several project actions to improve the conservation status of Mediterranean trout populations in pilot and transferability areas.

1. The artificial reproduction of wild pure spawners (selected by the above-mentioned “advanced” genetic analysis) in temporary or stable hatcheries, to produce genetically pure fertilized eggs and/or alevins for restocking and reintroduction activities. Each hatchery was designated for a specific basin corresponding to the origin of the breeders, which were to be captured annually from different selected locations.
2. Trout populations showing the highest levels of genetic introgression (approximately >80%) were selected for the total eradication of all alien Atlantic individuals through repeated electrofishing removal actions. The restoration of native populations, through reintroductions, started at the end of the eradication phases. To optimize effectiveness, eradication actions were carried out several times a year between August and November, when environmental and biological conditions facilitate catchability.
3. Trout populations characterized by moderate levels of genetic introgression (around 50%) were subject to experimental selective fishing. Based on the morphological distinction between alien and native phenotypes, specifically trained anglers should selectively remove alien specimens, hence improving the genetic integrity of trout populations over time [52]. Involved anglers were asked to collect a fin clip to genetically assess the effectiveness of this action.
4. Contrasting illegal stocking is a wide-range strategy based on the consideration that recent worsening of genetic integrity in wild Mediterranean trout populations could also be due to this bad practice. The aim was to create a mapping and recording system of the illegal stocking actions in the pilot areas and quantify potential impacts. The implementation of this action included the improvement of the surveillance system, also through the installation of camera traps in the sites selected for conservation actions.
5. Enhancement of freshwater habitats. In accordance with the Habitat Directive 92/43/EEC and the Water Framework Directive 2000/60/EC, this action was aimed at addressing the primary factors contributing to degradation of river ecosystems and biodiversity loss: river fragmentation, reduced water flow, and pollution. The Minimum Instream Flow (MIF) monitoring [53], and the selection and removal of physical and hydraulic barriers for each pilot area were included in the project. We georeferenced both natural and artificial physical barriers on field that interrupt river continuity and prevent the movement of fish fauna—only those preventing connectivity between native populations were selected for removal.
6. The project aimed to develop Guidelines for the conservation and management of the Mediterranean trout and its habitat, intending to extend this model to all Italian Natura 2000 sites and protected areas where trout conservation efforts are required. The process of creating these guidelines involved a participatory approach, which included organizing roundtable discussions across Italy and a public consultation initiative. These discussions engaged various national authorities and stakeholders, including the Ministry of Environment, regional governments, Basin District Authorities, as well as environmental, scientific, and sport fishing associations.

### 2.8. Production of Native Eggs and Alevins in Captive Condition

Putative pure wild spawners were caught in selected populations during the early reproductive season (approximately November–January, according to the areas), and individual tissue samples were collected for (advanced) genetic analyses. Each individual was univocally tagged using a Passive Integrated Transponder (PIT tag; Biomark^®^, Boise, ID, USA) for subsequent identification and transported to the assigned facility. The timing of spawners collection was planned to minimize their time in captivity while allowing the completion of genetic analyses.

Mature spawners passing genetic selection criteria were stripped after being anaesthetized to minimize stress, prevent injuries and facilitate handling. Eggs were fertilized using the dry method and then transferred to hatching troughs. Water incubation temperature was maintained around 10–12 °C, ideally as close as possible to that of the native sites. At the end of the breeding season, wild breeders were returned to the sites where they had been caught.

The release of eggs at the eyed stage was carried out using Vibert boxes or artificial nests (cocooning), while yolk-sac alevins were directly released into the streams (preferably in appropriate microhabitats, such as vegetated shallow waters near the banks) in small groups after being acclimatized to the local temperature. Methods for transport and releasing eggs and alevins are detailed in D’Antoni et al. [54].

## 3. Results

### 3.1. Environmental and Demographic Characterization of Wild Populations in Pilot Areas

**Figure 4:**
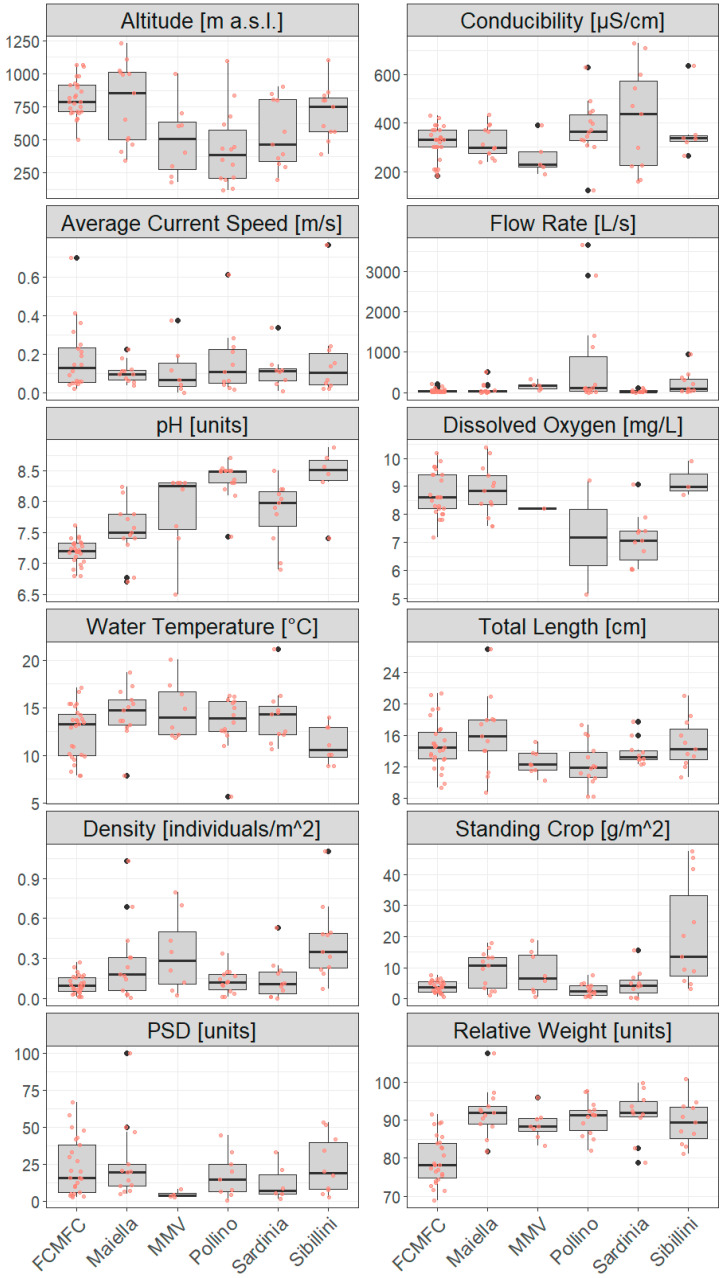
Boxplots of collected environmental parameters (units are indicated in square brackets) and demographic estimates for wild brown trout population across 84 sampling sites in six pilot areas. Rose dots indicate actual values.

Table 2 and Figure 4 summarize and display descriptive statistics of chemical-physical parameters and demographic measures (obtained from 5308 individuals) for each pilot area.

Except for water temperature and conductibility, we found overall statistical differences in all abiotic/environmental variables among examined areas (*p* < 0.004, 17.5 < H < 47.5; Kruskal–Wallis tests). Particularly, post hoc pairwise Mann–Whitney tests revealed that: FCMFC sites were at higher altitudes as compared to Pollino (*p* = 0.002, U = 48) and MMV (*p* = 0.04, U = 31); dissolved oxygen was lower in Pollino watercourses compared to FCMFC (*p* = 0.001, U = 22) and Maiella (*p* = 0.008, U = 11); pH was lower in FCMFC than in all other areas (*p* < 0.04, 1.5 < U <7 0.5), and higher in Pollino as compared to Maiella (*p* = 0.002, U = 11) and Sardinia (*p* = 0.03, U = 20.5).

Statistically significant differences were found among areas at all demographic parameters of trout populations (*p* < 0.03, 12.5 < H < 37.4; Kruskal–Wallis tests) except PSD (*p* = 0.15, H = 8.2; Kruskal–Wallis test). Statistically supported differences in median values between area pairs (Mann–Whitney tests) indicated: higher population density/standing crop in Sibillini than in FCMFC (*p* < 0.001 with U = 24, and *p* = 0.003 with U = 32, respectively) and Pollino (*p* = 0.01 with U = 16, and *p* = 0.003 with U = 9), and lower standing crop in Pollino compared to Maiella (*p* = 0.05, U = 30); substantially lower Wr in FCMFC compared to other areas (*p* < 0.03, 22 < U < 37).

**Figure 5:**
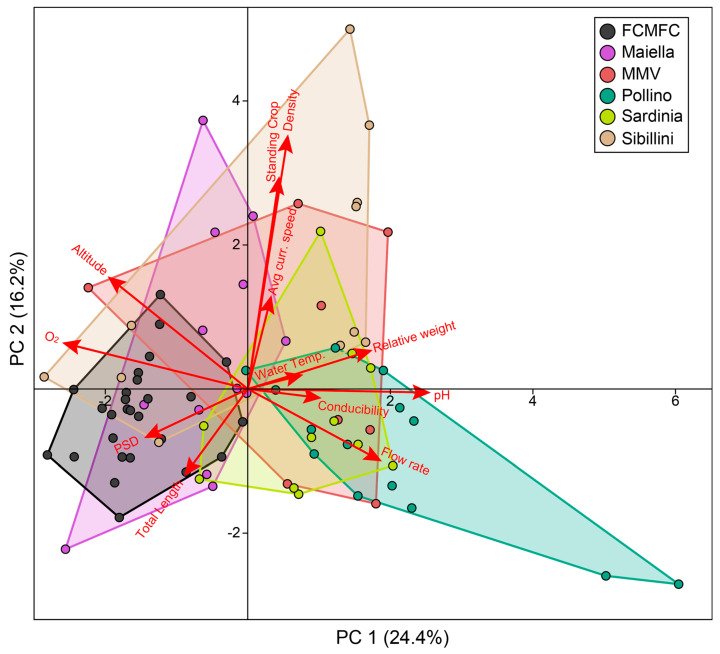
Principal components analysis based on (normalized) demographic and environmental features (red arrows) of 84 (Mediterranean) brown trout sampling sites (coloured dot).

The two first PCA axes accounted for approximately 40% of the total variability (Figure 5). The first axis showed a positive correlation with pH, water temperature, conductivity and relative weight, and a negative relationship with dissolved oxygen. Conversely, the second axis indicated a positive relationship with both population abundance/density and average water speed. The distribution of sampling sites in PCA confirmed substantial environmental and demographic diversification within (e.g., Pollino and Sibillini) and among (e.g., Pollino and FCMFC) the pilot areas.

### 3.2. Preliminary Genetic Characterization of Wild Populations

**Figure 6:**
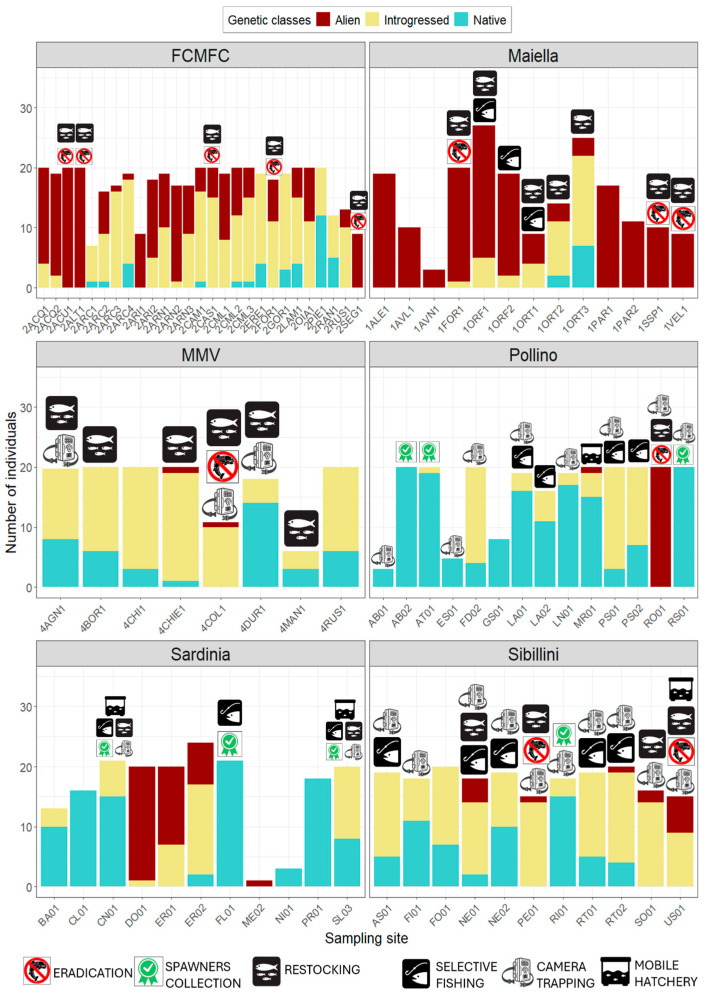
Bar charts depicting genetic class frequencies for 84 sampling sites of (Mediterranean) brown trout from six target areas in Italy. Selected sites for specific conservation actions (i.e., spawners collection, alien trout eradication, artificial reproduction in mobile hatchery, restocking with pure individuals, selective fishing, and camera trapping) were marked with symbols.

Preliminary genetic analyses involved a total of 1885 trout from 84 sites, revealing remarkably variable frequencies of genetic classes within and among areas (Figure 6). A relatively better conservation status emerged in Sardinia and Pollino where 12 watercourses appeared inhabited by native individuals exclusively (9 sites, including 4 with <10 sampled individuals) or at most (3 sites with 84–95% of native trout). A single less-introgressed population was found in Sibillini (83% native in RI01) and MMV (78% native in 4DUR1), while no native populations were found in FCMFC and Maiella. Despite efforts in sampling expansion, we could not find further wild (almost) native populations in MMV and Sibillini, while a single partly introgressed population was found in Maiella (Aterno) and FCMFC (Bidente di Pietrapazza). Hybrids/introgressed individuals occurred in 73.8% of the populations, being especially abundant in MMV and Sibillini. In total, 13 entirely alien populations (including 5 with <10 sampled trouts) were found across four pilot areas.

### 3.3. Advanced Genetic Characterization and Spawners Selection from Wild Populations and Hatcheries

After demographic and genetic cross-data checking, we could detect seven wild populations for spawner selection in three areas (three from Sardinia and Pollino, respectively, and one from Sibillini; Figure 6). To achieve planned conservation actions in the absence of wild populations that were sufficiently pure and/or demographically adequate, we had to integrate spawner stocks with genetically selected individuals from hatcheries rearing local Mediterranean trout. Specifically, we used spawners from the following:

- “Experimental Ichthyogenic and Hydrobiology Centre” of L’Aquila plus individuals from Aterno to supply Maiella populations.
- “Premilcuore” hatchery for FCMFC along with wild individuals from Fosso delle Cortine population (Bidente di Pietrapazza drainage).
- “Maresca” private hatchery (that maintained, on behalf of the Liguria Region, spawners from wild individuals caught in previous years in the local drainage of Val di Vara) plus few individuals from 4DUR1, Usurana and Malacqua rivers for MMV.
- “Borgo Cerreto” and “Cantiano” hatcheries—already successfully managed for local Mediterranean trout conservation actions within the LIFE19 IPE/IT/00015 IMAGINE and LIFE12 NAT/IT/000940 TROTA project frameworks, respectively, along with RI01 trout.

Advanced genetic analyses (results not shown) revealed clear differentiation among drainage basins in Pollino and Sardinia. Similarly, native hatchery stocks were well-differentiated, indicating affinities (i.e., shared D-loop haplotypes) with respective wild local populations.

Of 1431 putative spawners (including both wild and hatchery individuals), 948 were selected via advanced genetic characterization. The proportion of chosen spawners, relative to the total, showed considerable variation across the pilot areas, ranging from 46.7% in Maiella to 92.6% in Pollino (Table 3).

### 3.4. Reproduction of Wild Pure Spawners and Restocking Activities

Overall, up to the 2023–2024 mating season, 532,284 eggs were produced in five permanent hatcheries from FCMFC, Maiella, MMV and Sibillini, and two mobile hatcheries from Sardinia and one from Pollino (Table 3). Specifically, the permanent hatcheries produced 528,858 eggs, from which 60,500 eyed eggs and 137,594 alevins were released in 26 sites, while mobile hatcheries produced 3426 eggs, with 254 eyed eggs and 578 alevins released in four sites (Figure 5). In total, 130 restocking interventions were carried out.

### 3.5. Alien Trout Removal Activities and Native Trout Reintroduction

Twelve sites were chosen for the removal of the alien trout populations (Figure 6): 5 for FCMFC, 3 for Maiella, 2 for Sibillini and 1 for MMV and Pollino. Overall, 4627 alien trout were removed from 10 km of eradicated sites during 73 removal actions over four years (2021–2024; Table S1). After eradication, a total of eight reintroduction actions were carried out using 9772 eyed eggs and alevins, in addition to 149 translocated individuals (82 for Pollino and 67 for Sardinia) (Figure 6).

### 3.6. Selective Fishing and Actions Against Illegal Stocking

Overall, 150 trained fishermen were involved in selective fishing in 18 selected sites (including 1 extra site from the 84): 5 for Sibillini, 3 for Maiella and Sardinia, 4 for Pollino, 2 for FCMFC and 1 for MMV (Figure 5). Currently, a total of 225 samples have been collected from seven sites, 80.9% of which were Atlantic or hybrid/introgressed (Table S2). Fishermen and volunteer guards were also trained for site control to contrast the illegal stocking; wildlife cameras were installed in 23 sites for the same purpose.

### 3.7. Freshwater Habitat Improvement

To guarantee the conservation of wild and restocked populations, the yearly analysis of the MIF was carried out in all rivers stretches selected for concrete conservation actions. Physical barriers to be removed were selected, and we have foreseen three fish passes in FCMFC, two in Sibillini and Pollino, and one in Maiella.

### 3.8. Guidelines for the Conservation and Management of the Mediterranean Trout and Its Habitat

In 2023, the Guidelines were presented and discussed in four round table discussions held throughout the national territory. The final version of the Guidelines [54] took into account all of the comments submitted by the 400 stakeholders involved to create the most appropriate solutions.

## 4. Discussion

LIFE STREAMS is the first project aimed at the recovery and conservation of Mediterranean trout at the national scale, intending to provide a conservation management framework for the entire species range that would overcome issues associated with scarce coordination of conservation initiatives, collating and integrating the fragmented information available in the literature. The project primarily promoted a rationale to define site-specific conservation actions that relies on a genetic-demographic evaluation of the status of wild Mediterranean trout populations combined with an ecological assessment of biotopes.

In this extensive spatial framework, environmental characterization of sampling locations pointed out the typical habitat heterogeneity of small Mediterranean streams [55], to which native trout populations are adapted [56,57], and the lack of evident environmental issues that may preclude the applicability of conservation actions. Within a wide array of abiotic conditions, the dispersal processes of Mediterranean trout are highly influenced by (relatively cold) water temperatures [58] and average current speed, to which population abundance measures are related to (i.e., density and standing crop, Figure 5) [57]. For instance, the presence of a Sardinian trout population in the Flumineddu creek, where a summer temperature of 21.6 °C was recorded in stagnant water pools, corroborated observational data that has been reported by some authors for Sardinia [32,37] and other Mediterranean regions [59]. This tolerance to relatively extreme conditions, compared to the literature data for the brown trout, reflected the considerable plasticity of the species, as well as a possible local physiological adaptation to sub-optimal environmental features [60,61].

Recorded pH values were always alkaline, although significantly diversified among the target areas. As expected, the highest dissolved oxygen concentrations were observed for mountainous areas, i.e., Sibillini, Maiella, and FCMFC. The average water flow values were generally consistent and quite low across all regions, except for Pollino, where maximum measurements exceeded 3600 L s^−1^. In light of the torrential features of the rivers under examination, the restoration of river continuity and the preservation of MIF are of utmost importance for freshwater biodiversity conservation [15], also considering climate change predictions for the Mediterranean area [62]. This is a critical aspect of the research, as the long-term sustainable management of native trout populations could be compromised by the predicted increase in water temperature and the decrease in flow rates during drought periods, which would eventually cause limited habitat availability for trout and exacerbation of pollution phenomena [29,63]. In this context, removal of physical and hydraulic barriers should favour the recovery of ecological corridors and the connectivity of thermal refugia, potentially improving the resilience of Mediterranean trout populations [15]. To restore the river continuity and upscale protection of native fishes, six fish passes designed within the LIFE STREAMS project were earmarked to weirs downstream of which alien species were absent, to avoid facilitating their spread upstream.

Compared to the standard reference for the average areal biomass in salmonid watercourses [64], wild trout populations inhabiting the central Apennines (i.e., Sibillini and Maiella) were abundant (standing crop ≥ 10 g m^−2^), as opposed to other areas whose population abundances were below the carrying capacity of the watercourses (Table 2, Figure 3). Given the lack of limiting environmental conditions, this may be due to selective harvesting from angling, as suggested by low PSD values [15] that are generally below the 35–65 optimal range proposed by Gabelhouse [44]. These results provide essential suggestions to inform fishery regulation, in terms of minimum-length harvest limits [29] or the application of alternative fishing practices, such as the institution of “Catch and Release” areas, where fish are captured and immediately released [65]. The mean Wr values calculated for Maiella, Sardinia, Pollino, Sibillini, and MMV populations, were slightly below the optimal range of 95–105 units, reflecting quite good physiological conditions. In contrast, FCMFC populations notably indicated suboptimal body conditions, possibly mirroring the limited food availability in oligotrophic mountain creeks. Consistently, PCA supported increasing Wr at increasing water temperature and decreasing dissolved oxygen and altitude, thus capturing environmental changes predicted along the longitudinal profile of watercourses [66]. Besides this, an increasing trout abundance (standing crop/density) in more current waters, no strong relationship between demographic and environmental parameters emerged from PCA, indirectly corroborating the well-known ecologic plasticity of brown trout [67] and references therein. However, future in-depth analyses would evaluate possible ecological differentiation between native and exotic strains, as well as their hybrids.

The genetic conservation status of Mediterranean trout populations, although approximative because of the limited number of employed diagnostic markers, appears seriously compromised by genetic introgression from Atlantic hatchery strains in most wild populations, particularly in central and northern Apennines. Importantly, population-level assessments based on sole LDH-C1 provided similar outcomes to those obtained through microsatellites and diagnostic SNP panels, ensuring their overall reliability [68]. Such a pattern, previously denounced even within other protected areas in central-southern Italy (e.g., [14,17,27]), resulted from reiterate stocking with Atlantic alien individuals from hatcheries, both authorized and unauthorized, during the last century. Released alien trouts successfully survived and reproduced with native trouts (as proven by the widespread occurrence of hybrid-introgressed trouts; Figure 6), apparently giving rise to abundant (high standing crop and density values for Maiella and MMV; Figure 4) and/or age-structured populations in some cases (PSD > 35 in seven FCMFC sites; Figure 4). Notably, this has occurred despite the angling pressure and the interruption of stocking activities at least in protected areas. Besides this, detected genetically pure populations deserve special protection as genetic refugia, whose effectiveness in preserving/restoring native gene pools has been proven for brown trout populations in the eastern Pyrenean [69], although authors reported cases of (typical) genetic changes within populations due to genetic drift which were likely exacerbated by reduced population sizes [70,71].

Analyses conducted on the genetic population structure provided evidence for geographic-driven (strong) differentiation among watercourses within the Pollino and Sardinia areas, suggesting drainage basins as separate Management Units for Mediterranean trout conservation—consistently with outcomes from previous studies [6,17,37]. The same applies to more compromised and intricate contexts, such as FCMFC, where local gene pools appeared pervasively diluted by genes of Atlantic trout and even allochthonous Mediterranean trout, especially in the Tyrrenhian slope [47]. The study also suggested genetic differentiation among populations from Apennine slopes, hence indicating the Apennine chain as a barrier to trout dispersal, similarly to findings reported in Palombo et al. [27] for Mediterranean trout populations from central-southern Italy. In addition, a limited number of native/less introgressed (sub)populations were identified in an Adriatic basin (Bidente di Pietrapazza), which was consequently considered as a separate MU: restocking with native trout solely involved nearby populations within the same drainage. We followed the same approach in all pilot areas; however, it should be noted that such results are not shown as they fell outside the scope of the present study and will be published in dedicated papers. The reader may refer to Talarico et al. [47] as an extensive example of population structure analysis conducted within the FCMFC after sampling expansion.

All in all, our outcomes stress the importance of carefully identifying MUs within which we should be implementing conservation actions to preserve the Mediterranean trout’s local (genetic) polymorphism and distinctiveness [6,17,18,47,57].

### 4.1. Project Challenges

The project attempted to give a response to the urgent need for comprehensive and coordinated efforts to safeguard the Mediterranean trout while contextually providing a testbed to effectiveness of species management over a large scale. Flexibility in project design and conservation programs has been revealed as a strategic approach, allowing them to address unexpected circumstances and local issues. Here, we list major challenges emerging from project experience and possible solutions:

- The identification of native populations: The major issue was the (partly) unexpected absence of residual and viable native populations in many areas to sustain supportive artificial breeding. Given the general scarcity of pure-native populations, extended sampling may be provided for in some situations only, while spawner integration from hatchery stocks of Mediterranean trout should be minimized because of trout domestication and genetic diversity erosion [72,73].
- Translocations and supportive breeding: When pure-native populations are abundant and/or supportive breeding via hatcheries is inapplicable, translocations provide a more cost-effective approach for restoration of moderately depauperated populations. Conversely, to enhance partly introgressed and isolated wild populations for which genetically suitable and demographically adequate donor populations are lacking, any intervention could be avoided except for habitat protection, relying on the restorative power of natural selection [6,74]. Depending on the level of introgression, the institution of genetic refugees [71] may represent another practical conservation solution.
- Temporary vs. stable hatchery: Mobile hatcheries appear to be advantageous for minimizing spawners domestication and genetic diversity erosion through inbreeding [67,71], while reducing management costs. On the other hand, egg/alevin production was found to be much lower than in stable hatcheries, likely because of higher mortality and reduced adaptability to captive conditions of wild-caught spawners, coupled with their usually smaller size compared to hatchery-reared individuals [75]. As a suggestion for future research, the application of alternative techniques of semen cryopreservation, developed within the LIFE Nat.Sal.Mo. project [28] could be extremely useful in rationalizing breeding practices in hatcheries.

### 4.2. Future Perspectives

Monitoring and evaluating the effectiveness of adopted conservation measures –, e.g., through the quantification of introgression deviation in target populations after selective fishing, translocations and supportive breeding separately or in synergy, is a project task planned for 2025 that should provide essential information for future management. The limited duration of the project may hamper both the realization and/or the effectiveness validation of some long-term conservation measures. For example, obtaining authorizations for the removal of physical barriers has required much longer bureaucratic times than expected, making rescheduling necessary. Regarding selective fishing monitoring, preliminary genetic results on caught trout appear encouraging, considering the high percentage of Atlantic or hybrid/introgressed individuals removed by fishermen, although there is room for improvement of this conservation action. Moreover, it should be taken into account that the overall assessment of the fishermen’s ability to discriminate between Mediterranean and Atlantic/hybrid trout, which will be conducted in future studies, requires considering relative frequencies of genetic classes in target populations. The 5 years of “after LIFE” should guarantee monitoring and validation of the above-mentioned conservation actions and the improvement of the adaptive management approach. The long-term continuation of conservation action should be advantageously ensured by managers of protected areas.

## 5. Conclusions

This study highlights the relevance of a multilevel genetic–ecological approach for the conservation of intensively managed taxa, such as salmonids, which could be extended to other areas and species subject to similar management issues. Our data confirms pervasive alien introgression in most trout populations, resulting from intense stocking activities carried out over the last century for fishing purposes [6,76]. The National Guidelines for the conservation of the Mediterranean trout and its habitat [54] represent the most complete synthesis containing the information needed to undertake management policies and implement conservation projects for Mediterranean trout at different intervention scales. The involvement of all stakeholders, with particular reference to the large participation of fishermen in the round tables organized for the presentation of the Guidelines during the participatory process, represented a project strength, ensuring the long-term success of concrete interventions. Besides this, it should be taken into account that the outcomes attained through challenging conservation programs may be jeopardized by restocking practices. Although a derogation to the general ban on introducing non-native species into the wild (Decree of the President of the Italian Republic D.P.R. 357/1997), was introduced in Italy in 2019 (D.P.R. 102/2019), recreational anglers consider it too restrictive and requested a modification of the Italian derogation system in more permissive terms [20]. From our perspective, this aspect is of particular concern for the conservation of Italian native trout populations.

## Figures and Tables

**Table 1:** Summary of major climatic and environmental features for each of six pilot areas, along with previous information on the genetic status of local wild populations of (Mediterranean) brown trout and threats to which they are subject.

Pilot Area (Abbreviation)	Extension (ha)	Ecoregion	Elevation Range (m a.s.l.)	Climate	Prevailing Land Type	Main Hydrographic Systems	Background on Genetic Conservation status	Main Threats to Trout Populations
Foreste Casentinesi national Park (FCMFC)	36,843	Temperate	400–1658	Warm temperate–hot summers	Semi-natural forest habitats	Watercourses draining into either the Tyrrhenian or Adriatic Sea	Low occurrence of native trout [33,34]	Alien trout
Maiella National Park (Maiella)	74,082	Temperate	130–2793	Warm temperate–hot summers	Mountainous, Apennine deciduous and semi-deciduous forests	Orta, Orfento, Gizio (Aterno-Pescara Adriatic basin)	Pure and partially introgressed populations [35]	Alien trout, water abstraction, hydrological alterations
Montemarcello-Magra-Vara Natural Regional Park (MMV)	4597	Mediterranean	0–1639	Warm temperate–hot summers	Residual alluvial forests, rural areas	Vara and Magra rivers (Ligurian Sea)	Pure, almost pure and moderately introgressed trout populations [33,36]	Alien trout, water abstraction for agricultural purposes
Pollino National Park (Pollino)	192,565	Temperate	200–2000	Warm temperate–hot summers	Mountainous, minimal urban territory	River Sinni (Ionian basin), River Mercure-Lao (Tyrrhenian basin)	Pure Mediterranean trout (river Abatemarco) medium-low level of genetic introgression (River Lao) [13]	Water abstraction for agricultural purposes
2 SACs of Sardinia region: ITB041111, ITB011109, and 3 SPAs: ITB041105, ITB021103, ITB022212 (Sardinia)	138,873	Mediterranean	62–1829	Temperate with low level of continentality, prolonged Mediterranean summer droughts	Broad-leaved forest and natural grasslands. with a rugged landscape of deep valleys and rocky outcrops	Intermittently flowing Mediterranean rivers	Hybrid and native trout populations [37,38,39]	Alien trout, overfishing, water withdrawal, habitat fragmentation
Sibillini Mountains National Park (Sibillini)	71,400	Temperate	400–2476	Warm temperate–hot summers	Mountainous, Apennine deciduous and semi-deciduous forests	Watercourses draining into either the Tyrrhenian or Adriatic Sea	Residual nearly pure native trout populations [6]	Alien trout, river fragmentation, presence of hatcheries

**Table 2:** Summary (mean ± SD) of descriptive statistics for environmental parameters and demographic measures of wild trout populations, for six pilot areas.

Pilot Area	Elevation (m a.s.l.)	Conductivity (µS cm^−1^)	Average Current Speed (m s^−1^)	Flow Rate (L s^−1^)	pH (Units)	Dissolved Oxygen (mg L^−1^)	Water Temperature (°C)	Total Length (cm)	Density (ind m ^2^)	Standing Crop (g m^−2^)	PSD (Units)	Wr (Units)
FCMFC	814.3 ± 142.0	318.6 ± 68.8	0.18 ± 0.17	39.5 ±47.6	7.2 ± 0.2	8.7 ± 0.8	12.5 ± 2.8	14.77 ± 3.12	0.11 ± 0.07	3.91 ± 1.84	23.4 ± 19.1	79.47 ± 6.2
Maiella	776.7 ± 304.2	319.2 ± 64.7	0.11 ± 0.05	80.0 ± 142.7	7.5 ± 0.4	8.8 ± 0.9	14.4 ± 2.7	16.09 ± 4.75	0.28 ± 0.30	9.37 ± 5.79	25.8 ± 26.5	92.01 ± 6.3
MMV	503.1 ± 279.8	262.0 ± 78.5	0.12 ± 0.13	167.2 ± 102.3	7.9 ± 0.7	8.2	14.8 ± 3.0	12.54 ± 1.55	0.28 ± 0.30	8.27 ± 6.71	4.65 ± 2.55	88.73 ± 3.8
Pollino	433.1 ± 286.4	377.2 ± 114.4	0.16 ± 0.17	696.0 ± 1185.8	8.4 ± 0.3	7.2 ± 2.9	13.5 ± 2.8	12.27 ± 2.80	0.13 ± 0.09	2.82 ± 2.04	17.4 ± 14.7	90.51 ± 4.5
Sardinia	540.5 ± 254.8	414.4 ± 212.9	0.12 ± 0.10	28.4 ± 33.9	7.8 ± 0.5	7.1 ± 0.9	14.1 ± 3.0	13.87 ± 1.72	0.14 ± 0.15	4.79 ± 4.42	12.4 ±1 2.3	91.48 ± 6.5
Sibillini	704.9 ± 204.0	374.8 ± 131.8	0.17 ± 0.22	230.9 ± 281.9	8.4 ± 0.5	9.2 ± 0.6	11.1 ± 2.0	14.95 ± 3.08	0.42 ± 0.29	20.4 ± 17.0	24.2 ± 19.5	89.44 ± 5.9

**Table 3:** Summary information on spawner selection and artificial reproduction across pilot areas.

Protected Area	Years	Spawners	Genetically Suitable Spawners (Relative Ratio)	Fertilized Eggs	Released Material (Type)
FCMFC	2021, 2023	106	82 (77.4%)	6309	3994 (alevins)
Maiella	2021, 2022, 2023	638	298 (46.7%)	319,524	3500 (eggs), 5150 (alevins)
MMV	2021, 2022, 2024	181	139 (76.8%)	203,025	34,000 (eggs), 122,700 (alevins)
Pollino	2022, 2024	135	125 (92.6%)	1380	578 (alevins)
Sardinia	2022, 2024	146	105 (71.9%)	2046	254 (eggs)
Sibillini	2021, 2023	225	199 (88.4%)	n.a.	23,000 (eggs), 5750 (alevins)
Overall	2021–2024	1431	948 (66.2%)	532,284	60,754 (eggs), 138,172 (alevins)

## Data Availability

Data will be made available by the authors on request.

## Supplementary Materials

The following supporting information can be downloaded at: https://www.mdpi.com/article/10.3390/biology14050573/s1, File S1: Fish sampling and environmental data collection protocols, and Relative weight and PSD estimation methods; Table S1: Summary information on removal of alien Atlantic trout within target sites and pilot areas.; Table S2: Summary information on selective fishing activities for each pilot area during the project duration.

## Abbreviations

The following abbreviations are used in this manuscript:

IUCN	International Union for Conservation of Nature
MIF	Minimum Instream Flows
LDH-C1	Lactate Dehydrogenase chain-1 gene
SACs	Special Areas of Conservation
SPAs	Special Protection Areas
STREAMS	*Salmo ceTtii* REcovery Actions in Mediterranean Streams
PCA	Principal Components Analysis
