# Supplementary material for: The LIFE STREAMS Project for the Recovery of the Native Mediterranean Trout in Six Italian Pilot Areas: Planning and Adoption of Conservation Actions
Source: Biology (Basel). 2025 May 20;14(5):573. doi: 10.3390/biology14050573 (PMC12109421; doi:10.3390/biology14050573)
Supplement: Supplementary file 1 [file biology-14-00573-s001.zip › Carosi_etal-Supplementary File S1.pdf]

Supplementary material S1

Fish sampling and age determination protocols

A census of the fish fauna was carried out by electrofishing at each sampling location, using the two-pass removal method. Fish were captured during low flow periods using a continued or pulsed electric current with power varying between 600 and 4000W. The sampling was carried out in downstream to upstream direction for two consecutive times, applying the same fishing effort. Each site was sampled one time, and sampling was conducted during the morning. The length of sample sites was set as 10 times the mean width. The surveyed areas varied from a minimum of 100 m^2 to a maximum of 5,871 m^2 (mean ± SE = 529.30 ± 100.77). All captured fishes were identified and counted to estimate the density (individual m^{-2}) and standing crop (g m-2) for each population. For each individual, total length (TL, ±0.1 cm) and weight (±0.1 g) were measured. A sample of scales was collected from each trout for age determination. Approximately 10–15 scales were taken posterior to the dorsal fin and above the lateral line, on the left side of the body. When there were large numbers of sampled specimens, the scales were collected from a subsample for each increase in length of 1 cm to cover all length classes in the trout population. At the end of the field activities, all captured fish were released into their natural environment. The scales were stored in ethanol (30%) for later observation under a stereomicroscope using the image-analysis system IAS 2000. For each trout, age was determined independently by two observers, using the scalimetric method, and further validated by means of length–frequency distribution.

Environmental data collection

Conductivity (μS/cm), pH (units), water temperature (°C), and dissolved oxygen (mg/L) were measured at the same time as the fish samplings, using multiparametric probes manufactured by Hanna Instruments (Padova, IT). The hydrological parameters (flow rate (m^3 /s) and current speed (m/s) were measured at the cross-sectional area of each sampling reach, using an OTT MF-pro (Kempten DE) electromagnetic current meter (ISO 748:2007 Hydrometry _ Measurement of liquid flow in open channels using current-meters or floats).

Relative weight estimation

We assessed the relative weight (Wr) to estimate the body condition of trout. Wr was calculated using the following equation: Wr = 100 × (W/Ws), where W = body weight, and Ws = standard weight (i.e., the length-specific ideal biomass predicted by a length–weight regression calculated for a whole species to represent populations in better-than-average physiological conditions. The relative weight (Wr) is a condition index based on the comparison between the real weight of an individual and the optimal weight (Ws). Wr values lower than 95 indicate poor body condition. Wr estimation allows evaluation of the physiological status of fish, to compare specimens or populations of different lengths, and to highlight the occurrence of ecological changes over time. In the present study, the standard weight Ws was estimated using the following equation calculated for the S. trutta complex in the Tiber River basin using the empirical percentile (EmP) method, which is not influenced by the size of the specimens, as follows:

$\log_{10}$ (Ws)= −5.203+(3.154 log10 TL) −(0.015(log10 TL)^2)

where the TL application range (cm) is 8–58.

PSD estimation

We assessed the proportional stock density (PSD) index to provide a numeric estimation for deviations of the trout population structure from a balanced population. We calculated PSD using the following equation: PSD = 100 × (number of fish ≥ minimum quality length/number of fish ≥ minimum stock length). The minimum quality length was defined as the minimum size of fish that most recreational fishermen prefer to catch, while the minimum stock length was defined as the approximate length at sexual maturity. In this study, to establish the minimum quality length (TL = 25 cm) and the minimum stock length (TL = 22 cm), the values indicated by

Pedicillo et al. (2010) for the trout populations of Central Italy were used. The PSD values varied from 0 to 100, and the optimal range for a balanced population is $35 \leq PSD \leq 65$.
