# Supplementary material for: The LIFE STREAMS Project for the Recovery of the Native Mediterranean Trout in Six Italian Pilot Areas: Planning and Adoption of Conservation Actions
Source: Biology (Basel). 2025 May 20;14(5):573. doi: 10.3390/biology14050573 (PMC12109421; doi:10.3390/biology14050573)
Supplement: Supplementary file 1 [file biology-14-00573-s001.zip › Carosi_etal-Supplementary Table S1.pdf]

Supplementary material S2

Table S1

Summary information on removal of alien Atlantic trouts within target sites and pilot areas.

Protected areas	Years	Sites	# removed fished
FCMFC	2021, 2022	2CAS1	655
	2021, 2022	2ALT1, 2SEG1	328
	2022, 2023	2ACU1	1064
	2023, 2024	2FOR1	224
Maiella	2021	1FOR1	157
	2021-2023	1SSP1	1634
	2022	1VEL1	248
MMV	2021, 2022	4COL1	78
Pollino	2021-2023	RO01	33
Sibillini	2021, 2022, 2024	US01	142
	2021, 2022, 2024	PE01	64
**Overall**	**2021-2024**	**12**	**4627**
