# Supplementary material for: The LIFE STREAMS Project for the Recovery of the Native Mediterranean Trout in Six Italian Pilot Areas: Planning and Adoption of Conservation Actions
Source: Biology (Basel). 2025 May 20;14(5):573. doi: 10.3390/biology14050573 (PMC12109421; doi:10.3390/biology14050573)
Supplement: Supplementary file 1 [file biology-14-00573-s001.zip › Carosi_etal-Supplementary Table S2.pdf]

**Supplementary material S3**

**Table S2**

Summary information on selective fishing activities for each pilot area during the project duration.

| Protected areas | # sites | # catched fishes | Atlantic (relative ratio) | Hybrid/ introgressed (relative ratio) | Mediterranean (relative ratio) | n.a. (relative ratio) |
|---|---|---|---|---|---|---|
| FCMFC | 1 | 21 | 2 (9.5%) | 12 (57.1%) | 3 (14.3%) | 4 (19.0%) |
| Maiella | 3 | 161 | 115 (71.4%) | 18 (11.2%) | 1 (0.6%) | 27 (16.8%) |
| MMV | 1 | 7 | 0 (0%) | 4 (57.1%) | 2 (28.6%) | 1 (14.3%) |
| Sibillini | 2 | 36 | 0 (0%) | 31 (86.1%) | 5 (13.9%) | 0 (0%) |
| **Overall** | **7** | **225** | **117 (52.0%)** | **65 (28.9%)** | **11 (4.9%)** | **32 (14.2%)** |
